# Comparative analysis of complications and technique survival in peritoneal dialysis catheter insertion: single purse-string suture vs. double purses-string suture

**DOI:** 10.1080/0886022X.2024.2435209

**Published:** 2024-12-11

**Authors:** Xiaoling Li, Xiuling Chen, Hui Gao, Qin Zhou, Wenshu Liu, Pengli Li, Yan Li, Shiwen Wang, Jin Chen, Guisen Li

**Affiliations:** Department of Nephrology, Sichuan Academy of Medical Sciences and Sichuan Provincial People’s Hospital, School of Medicine, University of Electronic Science and Technology of China, Chengdu, China

**Keywords:** Peritoneal dialysis, catheter implantation, purse-string suture, complications, technical survival

## Abstract

**Objective:**

Single- and double-purse-string suture methods are both widely used in open surgical catheterization for peritoneal dialysis. This study aimed to compare the post-insertion complications and technical survival of the two methods.

**Methods:**

This retrospective study matched 142 patients who underwent peritoneal catheterization using the single- (Group S) or double- (Group D) purse-string suture method. Baseline clinical data and complications were recorded, and technical and patient survival rates were evaluated over 3 years.

**Results:**

There were no significant intergroup differences in terms of infection complication rates (S, 2.8% *vs.* D, 5.6%, *p* = 0.377) or non-infection complication rates (2.1% *vs.* 2.8%, *p* = 1.000) within the first month post-insertion. The Kaplan–Meier estimates of technical survival at 1, 2, and 3 years were 96.3%, 90.4%, and 85.9% in group S and 89.9%, 86.7%, and 84.8% in group D, respectively (log-rank test, *p* = 0.439). Additionally, patient survival rates were comparable between groups over the 3-year follow-up (log-rank test, *p* = 0.647).

**Conclusions:**

This study revealed that the single- and double-purse-string suture catheter insertion methods have similar post-insertion complication and technical survival rates. These data suggest that the single-purse-string suture method can be adopted as standard practice for peritoneal dialysis catheter placement.

## Introduction

Peritoneal dialysis (PD), the primary treatment modality for patients with kidney failure, is currently used widespread in clinical practice. An estimated 272,000 patients have undergone PD, representing approximately 11% of all dialysis patients globally [1]. In China, the number of patients has been increasing annually [2]. According to a report from the National Dialysis Quality Control Center, the number of patients with PD in China reached 140,544 by December 2022. However, the success of PD depends on the improvement in core outcomes, including PD-related infection, cardiovascular disease, technical survival, and patient participation. Complications related to PD catheter implantation affect technical survival [3,4]. A multicenter study in the United Kingdom found that 59% of patients (466 of 784) developed complications within the first year of catheter placement, resulting in 61.2% transitioning to hemodialysis [5]. Another study from the United States indicated that approximately one in nine patients developed infections or mechanical complications within 6 months of catheter placement [6].

PD catheter placement is crucial to successful PD access [7]. Open surgical techniques are widely used for PD catheter insertion. According to one survey, 70.3% PD centers in China use open surgical catheterization [8]. In the United States, 62% of PD centers offer the open surgical technique [9]. The advantages of surgical catheterization include its implementation under local anesthesia, execution by nephrologists, and reduced waiting time for surgery [10–12]. Studies indicate no significant differences in complications among the open surgical, laparoscopic, and Seldinger techniques [6,13–16]. Thus, open surgical catheterization remains highly relevant and useful in areas with rapidly increasing numbers of patients with PD. The classic open surgical technique involves inserting a PD catheter through the rectus abdominis muscle and fixing it to the peritoneum using pouch sutures. The reported methods include single-, double-, and three-purse-string sutures [17–22]. Some nephrologists believe that the single-purse-string suture method simplifies the operation and saves time [10,11]. Considering its potential advantages, the single-purse-string pouch suture technique has been used since 2011, whereas from 2007 to 2013, the double-purse-string suture technique was used in our center.

However, very few studies have compared complications of the single- and double-purse-string suture methods in peritoneal catheterization. Additionally, guidelines and reviews on PD access do not provide specific recommen­dations regarding these methods [10,12,23–25]. Thus, this study aimed to compare the differences in postoperative complications and technical survival rates of the single- and double-purse-string suture methods for PD catheter insertion. By analyzing these aspects, this study aimed to enhance our understanding of the single-purse-string suture method for PD catheter insertion.

## Materials and methods

### Study design and participants

This retrospective single-center case-control study was approved by the Ethics Committee of Sichuan Provincial People’s Hospital (no. 2017124). Informed consent for the open surgical technique was obtained from patients’ medical records. This study included patients who underwent PD catheter insertion between January 2008 and December 2017 at the PD center of Sichuan Provincial People’s Hospital. The inclusion criteria were as follows: (1) PD catheter implanted by open surgical technique; and (2) age > 15 years. The exclusion criteria were as follows: (1) catheter insertion using the laparoscopic or Seldinger technique; (2) PD not performed after catheterization; (3) diagnosis of acute renal failure; (4) transfer to hemodialysis or underwent kidney transplantation due to non-catheter-related complications within the first 3 months; and (5) history of abdominal surgeries, excluding appendectomy, laparoscopic cholecystectomy, and transverse cesarean section. The participants were followed up until December 31, 2020, dropout, or death.

### Groups

The patients were categorized into a single-purse-string suture group (group S) and a double-purse-string suture group (group D). In Group S, a single-purse-string suture with absorbable sutures was used to suture the peritoneal incision. In Group D, the peritoneum was sutured using two purse-string sutures. The standard straight catheters with double Dacron cuffs were used in all cases. The propensity score-matching method in SPSS version 26 (IBM, New York, USA) was used to calculate the propensity scores for both groups. The matching ratio for propensity score matching was 1:1, and the nearest neighbor matching method without replacement was used with a caliper width of 0.2 of the standard deviation of the logit of the propensity score. The variables included in the model were age, sex, Charlson Comorbidity Index, and operator. All surgeries were performed by four nephrologists specialized in PD. The operator referred to the same doctor as the matching criterion.

### PD protocol

On the third postoperative day, each patient underwent peritoneal lavage. If no catheter dysfunction was identified, PD was initiated. At our center, the initiation period for PD was 2 weeks post-catheter insertion before 2013 and was adjusted to 1 week post-catheter insertion thereafter. The PD protocol was 1 L of 1.5% PD solution administered three times daily, increased by 0.5 L every 3 days until a total volume of 2 L per exchange was reached. The final PD prescription was based on the patient’s residual renal function and peritoneal cavity volume to achieve a total Kt/V_urea_ ratio close to 1.7.

### Data collection

Baseline demographic, clinical, and biochemical variables including age, sex, cause of chronic kidney disease, Charlson Comorbidity Index, hemoglobin, serum albumin, corrected serum potassium, serum phosphorus, serum creatinine, serum urea nitrogen, intact parathyroid hormone, estimated glomerular filtration rate (Chronic Kidney Disease Epidemiology Collaboration formula), peritoneal equilibration test results, and Kt/V_urea_ ratio were recorded. All data were collected from the hospital information system.

### Events and definitions

An incision-site infection was defined as meeting one or more of the following criteria: 1) purulent drainage from the incision; 2) organisms isolated from a culture of fluid or tissue from the incision; and 3) swelling at the site, pain, or tenderness, redness, or heat.

A tunnel infection was defined as the presence of clinical inflammation (erythema, swelling, tenderness, or induration) with or without ultrasonographic evidence of fluid collection anywhere along the catheter tunnel.

An exit-site infection was defined as the presence of purulent discharge with or without erythema of the skin at the catheter–epidermal interface.

Peritonitis was defined as the presence of at least two of the following criteria: 1) clinical features consistent with peritonitis including abdominal pain and/or cloudy dialysis effluent; 2) dialysis effluent white cell count > 100/mL or > 0.1 × 10^9^/L (after a dwell time of at least 2 h), with > 50% polymorphonuclear leukocytes; and 3) positive dialysis effluent culture.

Catheter displacement was defined as the catheter’s location, as confirmed by radiography, being outside the pelvis.

Catheter obstruction was defined as blocked dialysate inflow and catheter outflow. This situation was considered resolvable with treatment.

Hemorrhage was defined as bleeding requiring transfusion or surgical intervention.

Leakage was defined as PD fluid leaking from the exit site during dialysis or imaging showing signs of fluid accumulation around the catheter.

Abdominal hernia was defined as umbilical and inguinal hernias.

Incisional hernia was defined as a hernia that developed at the site of the abdominal wall incision.

Events of death-censored technical survival were defined as any deaths unrelated to technique failure (such as heart disease, accidents, etc.) are considered and not included in the survival analysis of technique failure.

### Statistical method

All statistical analyses were performed using SPSS version 26 (IBM). The Shapiro-Wilk test was used to assess the normality of the variable distribution. Qualitative information is described as mean or median. T-tests were performed to analyze normally distributed variables, while the Mann-Whitney U test was used to examine non-parametric data. Chi-square tests were used to compare categorical variables between groups. Technical and patient survival data were summarized using Kaplan–Meier survival curves. The survival curves of the different groups were compared using the log-rank test. P values < 0.05 were considered statistically significant. All probabilities were two-tailed.

## Results

### Patient demographics and baseline characteristics

A total of 492 patients were screened for this study. After propensity score matching, 284 patients were enrolled in the analysis (142 in each group). The mean ages in the two groups were 47.4 ± 15.5 years and 50.0 ± 16.7 years, respectively, with 59.9% and 53.5% of the participants being male. The patients’ baseline characteristics are shown in Table 1.

**Table 1.** Baseline characteristics of the patients after propensity score matching.

Characteristics	Group S (*n* = 142)	Group D (*n* = 142)	X^2^/Z/F/t value	P value
Age (years)	47.4 ± 15.5	50.0 ± 16.7	−1.284	0.199
Gender (male)	85 (59.9%)	76 (53.5%)	1.162	0.281
Charlson comorbidity index	3.0 (2.0,5.0)	3.0 (2.0,5.0)	−0.833	0.377
Primary ESRD reason, *n* (%)			2.590	0.459
Chronic glomerular disease	75 (52.8%)	66 (46.5%)		
Diabetes mellitus	28 (19.7%)	27 (19.0%)		
Hypertension	23 (16.2%)	24 (16.9%)		
Other cause	16 (11.3%)	25 (17.6%)		
BMI (kg/m2)	21.0 (19.0, 23.1)	22.0 (19.8, 24.6)	−2.231	0.026
Operative times (minutes)	65.0 (55.0, 85.0)	90.0 (70.0, 114.0)	−6.713	0.000
Time of PD starting after insertion (days)	8.0 (7.0, 9.0)	11.0 (7.0, 16.0)	−4.670	0.000
eGFR((ml/(min/1.73^2^))	6.2 (4.7, 7.6)	5.7 (4.4, 7.3)	−1.450	0.147
Serum potassium (mmol/L)	4.4 ± 0.7	4.5 ± 0.8	−1.624	0.105
Serum albumin (g/L)	35.3 ± 6.4	37.0 ± 5.7	−2.268	0.024
Hemoglobin (g/L)	85.0 (79.0, 94.5)	86.0 (76.9, 96.0)	−0.311	0.756
iPTH (mmol/L)	333.0 (168.0, 508.0)	294.0 (177.5, 554.3)	−0.310	0.756
Total Kt/V	1.8 (1.3, 2.1)	1.6 (1.4, 2.2)	−0.065	0.948

ESRD: end stage renal disease; BMI: body mass index; eGFR: estimated glomerular filtration rate; iPTH: intact parathyroid hormone; Kt/Vurea: urea clearance rate.

### Complications within the first month post-insertion

Within 30 days of PD catheterization, there were no significant intergroup differences in the incidence of infectious or noninfectious complications (Table 2). Infection complications, including exit-site infections and peritonitis, occurred in 2.8% of patients in Group S versus 5.6% in Group D (*p* = 0.377). Noninfectious complications occurred in 2.1% of patients in Group S versus 2.8% in Group D (*p* = 1.000). Complications related to leakage and hemorrhage were extremely rare in both groups.

**Table 2.** Incidence of complications with the first month post-insertion.

	Group S (*n* = 142)	Group D (*n* = 142)	X^2^ value	P value
Infection complications, *n* (%)	4 (2.8%)	8 (5.6%)	1.392	0.377
Incision-site infection	0	1		
Tunnel infection	1	0		
Catheter exit site infection	2	4		
Peritonitis	1	3		
Noninfectious complications, *n* (%)	3 (2.1%)	4 (2.8%)	0.146	1.000
Catheter displacement	1	0		
Catheter obstruction	1	4		
Hemorrhage	0	0		
Intestinal perforation	0	0		
Leakage	1	0		
Abdominal hernia	0	0		
Incisional hernia	0	0		

### Complications within first year post-insertion

Over the first year, there were six and seven events of noninfectious complications in group S and group D, respectively. The rates of infectious complications were 11.3% in Group S and 23.2% in Group D. The infections of incision site, tunnel, catheter exit site were similar. Peritonitis was a primary cause of infectious complications (Table 3).

**Table 3.** Frequency (%) of complications within the first year post-insertion.

Event descriptor	Group S (*n* = 142)	Group D (*n* = 142)
Infection complications	16 (11.3%)	33 (23.2%)
Incision-site infection, *n* (%)	0 (0%)	1 (0.7%)
Tunnel infection, *n* (%)	1 (0.7%)	0 (0%)
Catheter exit site infection, *n* (%)	3 (2.1%)	4 (2.8%)
Peritonitis, *n* (%)	12 (8.5%)	28 (19.7%)
Noninfectious complications	6 (4.2%)	7 (4.9%)
Abdominal hernia, *n* (%)	1 (0.7%)	0 (0%)
Incisional hernia, *n* (%)	0 (0%)	0 (0%)
Leakage, *n* (%)	2 (1.4%)	0 (0%)
Catheter displacement, *n* (%)	1 (0.7%)	0 (0%)
Catheter obstruction, *n* (%)	2 (1.4%)	7 (4.9%)

### Complications in urgent start PD post-insertion

A total of 51 patients in Group S and 39 patients in Group D started PD within 7 days of catheterization. In Group S, only one patient developed peritonitis. In Group D, two patients experienced an incision infection and an exit site infection within the first month post-insertion (Table 4).

**Table 4.** Complications within the first month in urgent start PD post-insertion.

	Group S (*n* = 51)	Group D (*n* = 39)
Incision-site infection	0	1
Catheter exit site infection	0	1
Peritonitis	1	0
Noninfectious complications	0	0

### Technical survival and patient survival rates

**FIGURE 1.**
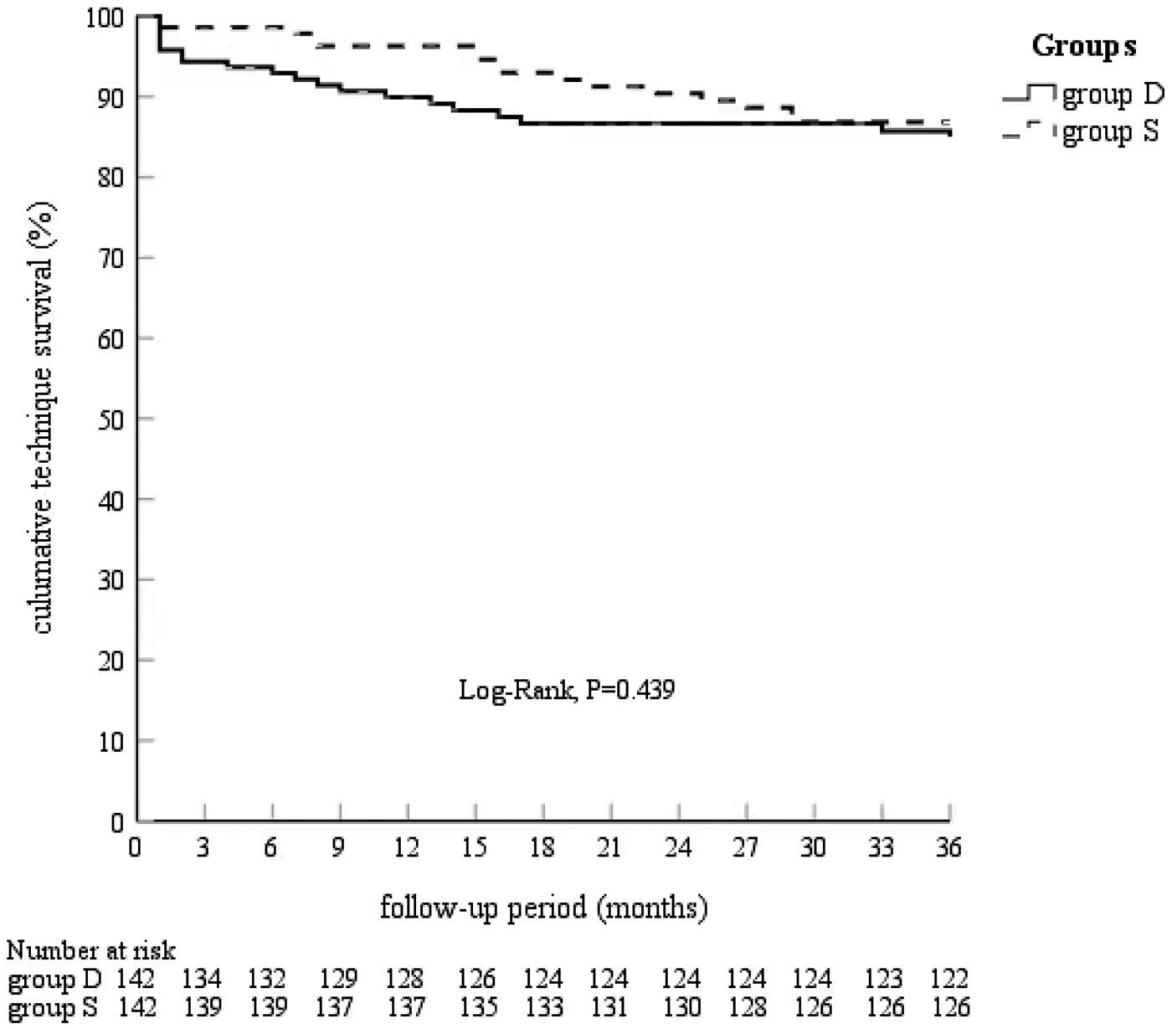
Cumulative technique survival stratified by placement technique.

**FIGURE 2.**
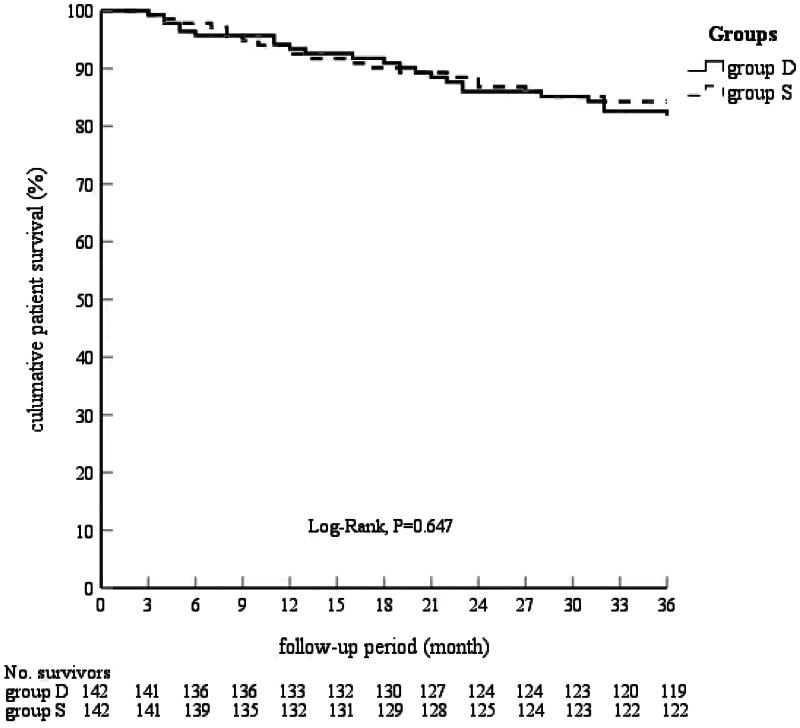
Cumulative patient survival stratified by placement technique.

The Kaplan–Meier estimates of technical and patient survival rates at 1, 2, and 3 years are shown in Figure 1. The technical survival rates were 89.9%, 86.7%, and 84.8% in Group D and 96.3%, 90.4%, and 85.9% in Group S, respectively (log-rank test, *p* = 0.439). The patient survival rates were 93.4%, 86.0%, and 81.7% in Group D and 92.5%, 86.8%, and 84.3% in Group S (log-rank test, *p* = 0.647), Figure 2. No significant intergroup differences were observed.

## Discussion

This retrospective study compared the complications and technical survival rates of PD catheter insertion using the single- and double-purse-string suture methods. In this study, no significant differences were observed between the two methods, suggesting that the single-purse-string suture method is feasible for PD catheter placement.

This study found that noninfectious complications in the first month and within the first year were comparable between the single- and double-purse-string suture methods. In the 2019 updated guidelines on peritoneal access in adults, the recommendations for the incidence of visceral injury (intestine, bladder, solid organs) and severe bleeding requiring blood transfusion or surgical intervention within 30 days of catheter insertion should be less than 1%, respectively [26]. The reported incidence rate of mechanical complications after catheterization ranged from 0.3 to 4.2% [6,17], catheter displacement being the most common complication [27]. The study results show that the single-purse-string method has a low non-infections complication rate, meeting guideline requirements and aligning with literature reports.

No difference in infection complication rates was observed between the two groups during the first month of catheterization. When we reviewed the 1-year complications, it appeared that peritonitis was more frequent in group D patients. No previous studies reported that different catheter insertion techniques affect the incidence of peritonitis. Previous studies have reported that the incidence of early exit-site infections and peritonitis after single-purse-string suture performed by nephrologists and surgeons ranged from 2.2% to 16.7% and 3.6% to 4.2%, respectively [17,25,28]. The incidences of early exit-site infection and peritonitis was 0.3% to 12.5% and 1.2% to 15.0% in patients with double-purse-string sutures [18,22,29]. According to peritoneal access guidelines, the incidences of exit-site infection/tunnel infections and peritonitis should remain below 5% within a month of catheter insertion [26]. A reported 25% of patients had catheterization failure, 6.5% had peritonitis, 10.6% had an outlet-site infection after 1 year of catheterization [5]. We speculate that there are two possible reasons for the higher incidence of peritonitis in group D. One reason is that the patients in Group D were our earlier cases, and we lacked experience to prevent and treat peritonitis during this period. Another reason is that factors contributing to the occurrence of peritonitis include general infection risks such as diabetes, frailty, and a high burden of comorbid diseases as well as specific peritonitis risks such as electrolyte imbalances, nasal carriage of *Staphylococcus aureus* [30]. Thus, the influence of surgical methods on the incidence of peritonitis becomes less significant after the perioperative period than during it. Consequently, it is challenging to directly compare the one-year incidence of peritonitis between Group D and Group S. Nonetheless, the peritonitis incidence in both groups close to the range reported in the literature. Compared to the studies mentioned above, we believe that the use of the single-purse-string suture method is associated with complications similar to those of the double-purse-string suture method.

There were no significant differences in leakage rates between groups S and D. Leakage is an early complication of catheterization. The incidence of leakage after catheterization varied from 0.1% to12.7% [22,31,32]. The double-purse-string suture method, which is widely used by PD centers in China, is considered more effective at preventing leakage than the single-purse-string suture method [11,32,33]. However, the double-purse-string suture method increases surgical complexity and procedural duration. Postoperative leakage is related to tightness of the purse-string suture and improper suturing of the fascia during catheterization [34]. Furthermore, the initiation of dialysis at an early stage may also contribute to leakage [35,36]. In this study, all patients started PD with low-dose fluids, which may have helped eliminate leakage events.

In this study, the technical survival rates were comparable between patients in groups S and D. The reported technical survival rates by open surgical technique varied across studies. A report from mainland China presented the survival rates of open surgical technique of 98% and 93% at 1 and 3 years, respectively [37]. An American study reported that 18% of patients had their catheters removed within 1 year of surgical catheterization [5]. A report from Taiwan stated that 35.4% of early start dialysis (dialysis after 1 day of catheterization) patients and 22.6% of late start dialysis patients (dialysis after 2 weeks of catheterization) in the first year after using single-purse-string suture method for catheter insertion [35]. A report from Hong Kong indicated 1- and 3-year technical survival rates of 92.7% and 87.2%, respectively [17]. The technical survival results in our patients were similar to those reported previously.

To the best of our knowledge, this is among the largest sample size studies comparing the single- and double-purse-string suture method for PD catheter implantation. This study also conducted a long-term follow-up to evaluate the possible effects of these two methods. However, it has several limitations. First, it was retrospective in size, which diminishes the robustness of the conclusions in comparison with those drawn from prospective trials. Second, this was a single-center study, and its conclusions require further validation in a multicenter setting. Finally, although propensity score matching was used to pair the participants, the two suture methods were performed at different times, which could potentially serve as a confounding factor for the higher incidence of peritonitis within the first month after catheter implantation in this study. Despite similar postoperative complications and long-term catheter survival rates, Group S had lower baseline body mass index and serum albumin levels and began peritoneal dialysis earlier than Group D. Although these differences may not significantly affect the overall judgment of the results, they do increase the potential for bias.

## Conclusion

In conclusion, this study revealed similarities in post-insertion complications and catheter technical survival rates between the single- and double-purse-string suture methods for peritoneal dialysis catheter implantation. These findings highlight the viability of the single-purse-string suture method, which simplifies the surgical process and is worthy of widespread clinical use.

## Data Availability

The data that support the findings of this study are available from the corresponding author upon reasonable request.
